# The Role of Pharmaceutical Compounding in Promoting Medication Adherence

**DOI:** 10.3390/ph15091091

**Published:** 2022-08-31

**Authors:** Maria Carvalho, Isabel F. Almeida

**Affiliations:** 1 Professional Compounding Centers of America (PCCA), Department of Research & Development, Houston, TX 77099, USA; 2 UCIBIO-REQUIMTE, MedTech, Laboratory of Pharmaceutical Technology, Department of Drug Sciences, Faculty of Pharmacy, University of Porto, 4050-313 Porto, Portugal; 3 Associate Laboratory i4HB—Institute for Health and Bioeconomy, Faculty of Pharmacy, University of Porto, 4050-313 Porto, Portugal

**Keywords:** geriatric formulations, medication adherence, patient-centric design, pediatric formulations, pharmaceutical compounding, pharmaceutical intervention, polypharmacy

## Abstract

Pharmaceutical compounding is an important component of pharmacy practice despite its low prevalence. Several therapeutic needs can be met by a compounded medicine such as dosing adjusted for pediatric patients, special drug combinations, medicines for patients allergic to a given excipient, and medicines for orphan drugs not provided by the pharmaceutical industry. Examples of such applications are provided in this review. Adherence to medication is a critical public health issue as nonadherence to pharmacotherapy has been associated with adverse outcomes and higher costs of patient care. Adherence to therapy represents a key factor in the reduction in morbidity and mortality and optimization of the use of financial resources. The role of pharmaceutical compounding in promoting medication adherence is underexploited. The customization might represent a positive reinforcement of the initiation of the treatment, while implementation and persistence might also be favored in a pharmacy setting. However, studies addressing the influence of compounding in adherence promotion are lacking in the literature. The results of such studies could support health policies including proper regulatory framework, pharmacist training, and information to health care practitioners.

## 1. Introduction

Medication adherence has been defined as an active, cooperative, and voluntary participation of the patient in following recommendations from a healthcare provider regarding dosing regimens [1]. This behavior can be further divided into three different steps: initiation (when the patient takes the first dose); implementation (the extent to which the prescribed dosing regimen is followed by the patient); and persistence (the period until treatment discontinuation) [1]. Non-adherence is an increasingly relevant public health issue. In Europe alone, it has been shown to contribute to nearly 200,000 premature deaths, resulting in costs of EUR 125 billion per year in avoidable hospitalizations, emergency care, and outpatient visits [2]. Multiple factors can influence medication adherence. The World Health Organization classifies the causes of non-adherence into five main dimensions: socioeconomic factors, health care and system-related factors, therapy-related factors, condition-related factors, and patient-related factors [3]. Several studies have shown that adherence to pharmacological treatment is far from expected, pointing to a rate of 50% of non-adherent patients [4] which can reach 75%, as described for psoriasis patients using topical treatments [5]. Several therapy-related factors can influence adherence, including drug product characteristics such as swallowability, packaging, dosing regimen, container closure system, and type of dosage form [6]. For instance, orodispersible dosage forms present the advantages of being easy to swallow without drinking or chewing and they do not require water for patient administration [7]. Patient adherence is generally improved in comparison with conventional solid dosage forms. One such example is the administration of olanzapine as an orodispersible tablet compared with conventional tablets [8].

Patient preferences for a given vehicle can also vary according to the disease [9]. For patients with acne, a recent study showed that a tretinoin lotion was preferred over the cream [10] while for psoriasis creams, ointments, and foams (particularly for the scalp) were appointed as the preferred dosage forms [9].

Recently, the role of the vehicle in treatment adherence has been objectively evaluated in a sample of psoriasis patients [5]. Adherence was significantly higher for patients applying gels and creams than for those using ointments, whenever the lesions affected an extensive body area. These outcomes, mediated by the dosage form, reinforce the relevance of a proper design of pharmaceutical formulations to effectively address the public health problem of non-adherence and paves the way for a more prominent role of pharmaceutical compounding on treatment adherence improvement.

## 2. Pharmaceutical Compounding and Regulatory Framework

Pharmaceutical compounding corresponds to the preparation of customized medicines in order to meet the specific needs of patients, which cannot be met by the commercially available medicines provided by the pharmaceutical industry [11]. This is an age-old practice in which pharmacists combine, mix, or alter ingredients to create unique compounded medicines based on the doctor-patient-pharmacist (triad) relationship. Compounding is currently an integral part of pharmacy practice and it is essential to the provision of healthcare. The current COVID-19 pandemic has highlighted the importance of compounding where pharmacists worldwide were able to promptly prepare hand sanitizers and surface disinfectants; oral liquids and capsules including hydroxychloroquine sulfate, dexamethasone, remdesivir, and other potentially promising drugs prescribed off-label. It is estimated that compounded medicines represent between 1% and 3% of pharmaceutical prescriptions and their use is growing [12]. The increasing need for compounded medicines is likely to be related to numerous factors, such as: limited dosage forms; limited dosages/strengths; limited orphan medicines; need for alternative raw materials and organoleptic characteristics; need for special combinations of active pharmaceutical ingredients (APIs); shortages and discontinued commercial medicines. Compounding is an important therapeutic option in all areas of medicine, with particular relevance in the pediatric and geriatric specialties which are often underserved by the pharmaceutical industry (Section 3). Although compounded medicines do not require regulatory approval, the practice of pharmaceutical compounding falls under the jurisdiction of national or state boards of pharmacy. The regulatory framework is not globally harmonized and, as a result, the standards of practice, compounding settings and definition vary considerably across the United States of America (USA) and the European countries. Strict compliance to the regulatory framework is essential to the provision of compounded medicines with the required quality, safety, and efficacy. Pharmaceutical compounding errors are preventable but, unfortunately, there are reports of unintentional errors that have caused serious harm to patients worldwide. The most impactful error dates back to 2012 when contaminated steroid injections compounded by the New England Compounding Center (NECC) were administered to 14,000 patients, leading to a multistate outbreak of fungal meningitis with over 60 reported deaths [13]. This error in particular was the result of microbiological contamination but there are other categories of errors potentially as harmful. For instance, administration of compounded medicines with the incorrect ingredients or the incorrect dosage strengths [12,14].

Pharmaceutical compounding is safe and essential, provided that all these categories of errors are anticipated and prevented by following the compounding regulatory framework.

### 2.1. Compounding Regulatory Framework in the European Union

The preparation of pharmaceutical drug products in pharmacies is not harmonized across Europe although there are some common definitions. Compounding activity is mainly regulated at the national level. Compounding formulations were first defined by Directive 89/341/CE and maintained in the Directive currently in force (2001/83/CE). Two categories can be distinguished:

i. magistral formula: any medicinal product prepared in a pharmacy in accordance with a prescription for an individual patient;
ii. official formula: any medicinal product which is prepared in a pharmacy in accordance with the prescriptions of a pharmacopoeia and is intended to be supplied directly to the patients served by the pharmacy in question.

Since the 7th edition, European Pharmacopeia (EP) included a monograph of pharmaceutical preparations [15,16] that makes a distinction between preparations for whom formal licensing is required and preparations exempt from licensing (which encompass compounding formulations). According to EP, pharmaceutical preparations may be licensed by the competent authority or unlicensed and compounded to meet patients’ specific needs. There are two categories of unlicensed pharmaceutical preparations:

i. extemporaneous preparations, i.e., pharmaceutical preparations individually prepared for a specific patient or patient group, supplied after preparation;
ii. stock preparations, i.e., pharmaceutical preparations prepared in advance and stored until a request for a supply is received.

Risk assessment should be undertaken for unlicensed preparations by identifying the criticality of different parameters and the risk the preparation may present to a particular patient group.

### 2.2. Compounding Regulatory Framework in the United States

The definition of pharmaceutical compounding in the USA is not harmonized across the Drug Quality and Security Act (DQSA), the US Food and Drug Administration (FDA), the United States Pharmacopeia-National Formulary (USP-NF) and the National Association of Boards of Pharmacy (NABP). Although similar, these definitions do not necessarily include all the same activities in the term compounding [17]. In accordance with the USP-NF Chapter <795>, compounding is defined as “the preparation, mixing, assembling, altering, packaging, and labelling of a drug, drug-delivery device, or device in accordance with a licensed practitioner’s prescription, medication order, or initiative based on the practitioner/patient/pharmacist/compounder relationship in the course of professional practice” [18]. The laws, regulations, standards, and guidelines for pharmaceutical compounding in the USA have been suffering significant changes since the NECC tragic compounding incident. As a direct result of this outbreak, the DQSA (H.R.3204, 113th Congress, 2013–2014) was signed into law which amended the Federal Food, Drug and Cosmetic Act (FFDCA) with respect to the regulation of compounding drugs (Title I: Drug Compounding—Compounding Quality Act) and drug supply chain security (Title II) [19]. In summary, the Compounding Quality Act exempts compounded drugs from new drug requirements, labeling requirements, and track-an-trace requirements; establishes requirements for pharmacies and outsourcing facilities under the sections 503A and 503B, respectively; and requires the publication of a list of drugs difficult to compound, among other amendments [20]. As such, there are now two types of compounding facilities in the USA which are subject to different regulations:

i. Pharmacies (section 503A): State-licensed pharmacies that traditionally prepare compounded medicines for a specific patient prescription and that are overseen by the State Boards of Pharmacy.
ii. Outsourcing facilities (section 503B): a new type of designation for those compounding facilities that stand in between the traditional compounding pharmacies and the large pharmaceutical manufacturers. These facilities are not required to be licensed pharmacies but must be supervised by a pharmacist or physician. Importantly, all 503Bs must comply with the current good manufacturing practices (cGMP) and undergo regular FDA inspections on a risk-based schedule. In addition, all products compounded, which may or may not be patient-specific, must be reported to the FDA every 6 months [19]. As of 15th February 2021, there were 69 facilities registered as human drug compounding outsourcing facilities in the USA [21].

Standards of quality for compounded and manufactured pharmaceuticals have been developed by the United States Pharmacopeial Convention, a nongovernmental and private organization, and are published in the USP-NF, as well as in the corresponding USP Compounding Compendium [22]. The key USP-NF chapters that relate to the practice of pharmaceutical compounding are as follows: <795> Pharmaceutical Compounding—Nonsterile Preparations [18] <797> Pharmaceutical Compounding—Sterile Preparations [23]; and <800> Hazardous Drugs—Handling in Healthcare Settings [24]. Although official compendia, the USP-NF has no enforcement powers and the individual State Boards of Pharmacy may or may not adopt these compounding standards. Currently, some State Boards of Pharmacy are actually modifying or developing and implementing their own standards for sterile and nonsterile compounding [8].

## 3. Compounding Formulations Addressing Patient Needs and Preferences

Drugs do not work in patients who don’t take them (C. Everett Koop, MD, US Surgeon General, 1985). When the reasons behind medication nonadherence are well understood by the triad relationship (doctor–patient–pharmacist), the preparation of a patient-specific medicine may be the solution to improve treatment adherence. There are many reasons why patients frequently make the rational decision not to take their medication as prescribed, in particular the pediatric and geriatric special populations. Medication adherence in pediatrics is often a challenge because children either do not want or are unable to take solid dosage forms such as tablets and capsules. Furthermore, children are very sensitive to the taste and smell of medications and if the patient’s preferences are not taken into account, it is very likely that the medication will be refused. Parents and caregivers often struggle to give medication to children and treatments get compromised when dosages are missed [25]. Medication adherence in geriatrics is even more complex because physical, emotional, and social issues influence the elderly’s decision making. In addition, many geriatric health problems are chronic rather than acute, so medications are often taken for life [8]. If patients are not fully satisfied with the prescribed medications, it is very likely again that dosages are missed, and treatments compromised. When there is a lack of communication, physicians may needlessly increase the medication dosage to achieve the desired efficacy, resulting in potential harm to patients and unnecessary costs overall. Taking into account that the aging population is growing and that life expectancy is increasing, tackling medication nonadherence is deemed a priority in today’s healthcare.

Pharmaceutical compounding may be key to addressing these age-related issues and ensuring treatment adherence. Some compounded formulations illustrating potential solutions, spanning dosage forms for different administration routes as well as flavoring and packaging options, will be addressed below.

### 3.1. Special Patient Populations

#### 3.1.1. Pediatric Patients

##### Age-Appropriate Dosage Strengths

The pediatric population is very different from adults and corresponds to a heterogeneous group, ranging from preterm newborn infants to adolescents, with specific therapeutic needs throughout growth and development. In pediatrics, dosage strengths are usually calculated based on the age, body weight (mg/kg), or surface area (mg/m^2^) of the children. For instance, a 3-year-old toddler with 15 kg requires 10 mg once daily of the proton pump inhibitor omeprazole for the treatment of benign gastric ulcers, whereas a newborn of 4 kg requires only 2.8 mg once daily (700 micrograms × 4 kg) of oral omeprazole [26]. Taking into account that the majority of commercial medicines are available in standardized dosage strengths, intended for adults, access to age-appropriate dosage strengths is critical to providing safe and effective pharmacological treatments to children [11,27].

##### Child-Friendly Dosage Forms

**Figure 1:**
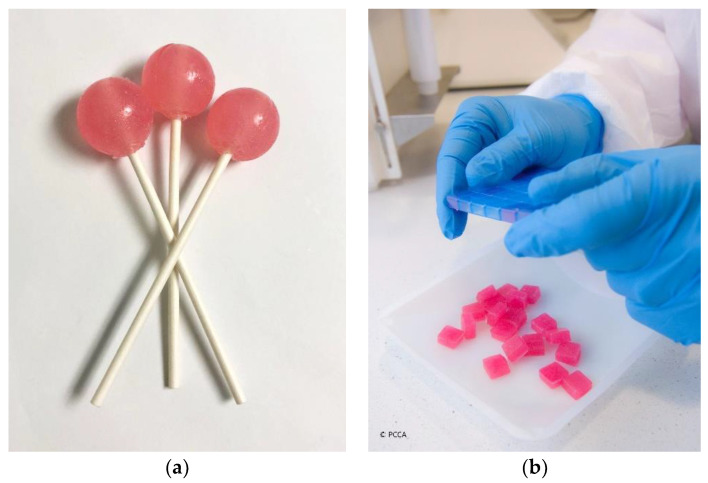
Child-friendly dosage forms: (**a**) medicated lollipops; (**b**) medicated lozenges (troches) (courtesy of PCCA, 2021).

Oral liquids, including emulsions, solutions, and suspensions, are the most commonly used dosage forms in pediatrics. However, the taste, smell, appearance, mouthfeel, and volume of liquid taken at each dose may be extremely odious to some patients. An alternative option to oral liquids is child-friendly dosage forms, such as medicated lollipops and lozenges (troches) [28,29], as illustrated in Figure 1. Administration of these dosage forms is facilitated because they dissolve slowly in the mouth or can be chewed and swallowed easily. Soft and chewable lozenges may be novelty-shaped (e.g., gummy bears) which makes them even more attractive. These unconventional but promising dosage forms may also be flavored to suit taste preferences and colored to entice the most reluctant pediatric patient [30]. A sample formula for anti-gag lollipops is shown in Table 1. Pharmacists may choose to prepare placebo taste samples of lollipops and lozenges for children so that they have the option to select their medication and be an integral part of the decision making [12]. Medication adherence is considerably improved when children get actively involved in their own treatments. A limitation of these child-friendly dosage forms is that they get mistaken for candy. Caution must be made to always keep medication out of reach and sight of children.

##### Flavoring and Sweetening

The importance of taste masking in pediatrics is undisputed. If younger patients dislike the taste, smell, and/or appearance of their oral medications, adherence to therapy will undoubtedly be at risk [31]. Commercial oral liquids are formulated taking into account the preferences of the general population but ‘one size does not fit all’—patients are individuals, respond as individuals, and must be treated as individuals [12]. This is when pharmaceutical compounding plays a major role in medication adherence. Pharmacists may prepare alternative compounded medications including the right combination of flavoring agents and sweeteners to match the patient’s preferences. The FLAVORx pediatric system is an example of the wide range of flavors currently available for children that may be used in pharmaceutical compounding. Upon subscription, pharmacists have access to an online comprehensive flavoring formulary with thousands of recipes including flavors, sweetening enhancers, and bitterness suppressors [32]. Usage data from 2011 has shown that the preferred flavors of U.S. consumers are grape, bubblegum, strawberry, watermelon, and cherry [33].

#### 3.1.2. Geriatric Patients

##### Polypharmacy

The elderly often live with multiple health conditions (multimorbidity) that may require medication to tackle each condition individually, leading to a high drug intake (polypharmacy) on a regular basis. The prevalence and incidence of polypharmacy, as a result of multimorbidity, is considerably high in geriatrics and may contribute to medication nonadherence due to the significant burden on the patient’s lives. Pharmaceutical compounding may reduce the medication regimen complexity by providing special drug combinations that bring together several commercial medicines in one single dosage form. Oncology and pain management are among those medical specialties that benefit the most from drug combinations. For instance, compounded mouthwashes for chemo-induced oral mucositis (CIOM) play a major role in the quality of life of cancer patients. These mouthwashes usually combine diphenhydramine, aluminum-magnesium antacids, and lidocaine, with antibiotics such as nystatin or tetracycline to prevent/treat infections; corticosteroids may also be added to help with the mucosal inflammation in immunocompromised patients [34]. An example of a compounded mouthwash for CIOM including multiple ingredients (Stanford modified oral rinse) is shown in Table 1.

Transdermal medications allow different types of drugs, in various dosage strengths, to be delivered simultaneously in one topical application. In addition, these permeation-enhancing medications bypass the hepatic first-pass metabolism, allowing for greater bioavailability and decreased dosing to achieve therapeutic effects [35]. An example of a special drug combination in pain management is shown in Table 1, which includes ketamine, gabapentin, clonidine, and baclofen in a permeation-enhancing proprietary vehicle. This formula has been demonstrated to facilitate the effective delivery of all ingredients through human skin and it is most beneficial in pain management because of the multiple receptors and pain pathways that can be targeted simultaneously [36].

##### Dysphagia

Dysphagia, physical and/or psychological swallowing difficulties, is estimated to affect 1 of 6 adults in the USA [37]. Patients at the greatest risk of developing dysphagia include those with degenerative neurological or muscular disorders, such as Alzheimer’s and Parkinson’s diseases, which have a higher incidence in geriatrics. Stroke, oropharyngeal tumors, head/neck injury, or stroke also put patients at a higher risk of developing dysphagia. This disorder may have a significant impact on the patient’s quality of life as it affects not only the pharmacotherapy but also nutrition and hydration, with potentially psychological and social effects [38].

Elderly dysphagic patients require alternate dosage forms to the commonly prescribed, difficult-to-swallow commercial tablets and capsules. Examples include oral disintegrating tablets, transdermal, rectal, and buccal drug delivery systems, and altered-thickness oral liquids [38]. Pharmaceutical compounding may provide patients with a variety of these alternate dosage forms to promote medication adherence and ensure treatment adherence. An example of a transdermal medication has already been addressed as an alternative to the pill burden in pain management. An additional example is shown for a budesonide mucoadhesive oral suspension, a slightly thick liquid that may be helpful to patients who are susceptible to think liquid aspiration (Table 1).

##### Packaging

Functional and cognitive impairment in the elderly may affect their ability to self-administer medication. Common struggles include opening containers with a lid, removing medication from blisters, and cutting tablets to get the target dose. If these impaired patients do not get the necessary help when it is medication time, nonadherence is likely to compromise the outcome of their treatments [39].

An array of compliance packaging solutions have been designed with the aim to promote adherence by simplifying the administration of medication to geriatric patients. Pharmacists in the community setting are now able to repack commercial tablets and capsules in multidose dispensing systems (MDDS). These versatile customized packs also simplify medication management by organizing the pill burden in monthly or weekly calendar layouts [40].

**Figure 2:**
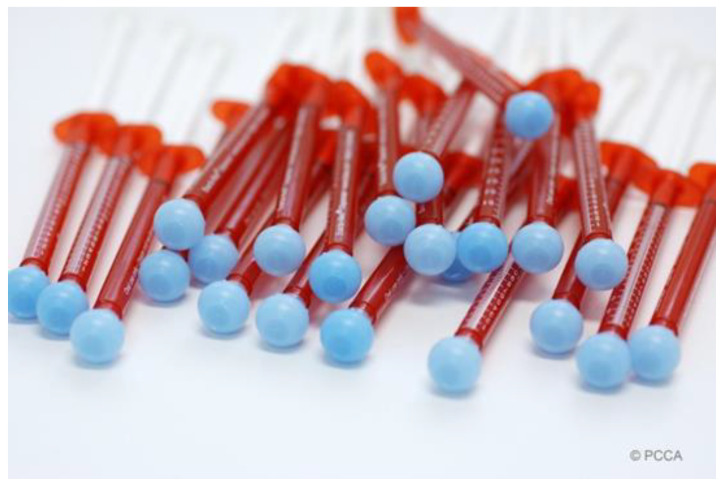
Single-dose syringes (courtesy of PCCA, 2021).

If patients take compounded medications instead, which are customized to meet their individual needs, the medication packaging should be intentionally user-friendly. For those patients who struggle opening containers, multidose oral liquids may be dispensed in single-dose oral syringes (Figure 2). When the manual division of tablets represents a problem, the right dosage may be individualized in capsules [41]. For those patients suffering from dysphagia (Section 3.2.2), pharmacists may use Coni-Snap^®^ Sprinkle capsules so that patients can easily and safely open the contents of the capsules due to their innovative closure system [42]. The uniqueness of pharmaceutical compounding is that it enables pharmacists to be creative and, by working closely with their patients and physicians (triad relationship), the right medication in the right packaging may be dispensed at all times.

### 3.2. Patient-Specific Needs

#### 3.2.1. Allergies and Intolerances

There is a growing need for alternative excipients in medications due to allergies and intolerances by sensitive patients. Potential troublesome excipients commonly seen in commercial medications include dyes, flavors, sweeteners, preservatives, gelatin, alcohol, milk products, and products containing gluten, corn, soy, or nuts. If a patient does not tolerate well the pharmacological treatment, adherence to medication will likely be affected. The solution to this problem is the preparation of an alternative compounded medication that excludes or substitutes the troublesome excipient(s). For instance, substituting lactose, a popular diluent in commercial tablets, for compounded capsules including a cellulose derivative, such as methylcellulose, in lactose intolerant patients. Likewise, substituting commercial gelatin capsules for vegetable-based compounded capsules consisting of starch or polymer, in vegetarian patients or those with religious restrictions against the consumption of pork products [43]. Pharmaceutical compounding is, almost always, the only available option for these sensitive patients and it therefore plays a key role in promoting medication adherence.

#### 3.2.2. Orphan Medicines

Medicines indicated for the diagnosis, prevention, or treatment of conditions with very low prevalence (rare diseases) are entitled ‘orphan medicines’. Currently, there are over 200 orphan medicines registered in Europe but it is estimated that there are between 5000 and 8000 different rare diseases [44]. In the absence of a licensed orphan medicine, rare disease patients rely exclusively on pharmaceutical compounding to fulfill their therapeutic needs. In these cases, orphan ‘compounded’ medicines are the only treatment option available. Examples of compounded medications prescribed for rare conditions are L-carnitine solution (for carnitine palmitoyl transferase1A deficiency), sodium thiosulphate injection (for calciphylaxis), and chenodesoxycholic acid capsules (for cerebrotendinous xanthomatosis) [45,46]. Furthermore, licensed orphan medicines are not always adequate for each and every rare disease patient. Again, ‘one size does not fit all’ and customized orphan ‘compounded’ medicines are likely to further promote medication adherence. For instance, the extemporaneous preparation of a sildenafil oral liquid instead of dispensing the licensed tablets (Revatio^®^) for pediatric patients with pulmonary arterial hypertension [45].

### 3.3. Other Specific needs

#### 3.3.1. Medicines Shortages

Occasionally, the pharmaceutical industry is not able to meet the demand for particular medicines, and commercial medicines become temporarily unavailable. Limited production capability, manufacturing problems, and lack of raw materials are some of the common causes of shortage of medicines. A current example is the shortage of hydroxychloroquine due to the sudden demand to treat COVID-19. Patients with rheumatic disease, for instance, who rely on a regular supply of hydroxychloroquine are now struggling to access this critical medication [47]. In these situations, pharmaceutical compounding is a valuable resource to “bridge the gap” until commercial medicines are once again widely available [11]. An example of a compounded hydroxychloroquine oral suspension is shown in Table 1.

#### 3.3.2. Discontinued Medicines

There is a long and growing list of proprietary medicines that have been discontinued by the pharmaceutical industry, mainly for commercial reasons. As a result, patients may no longer access important commercial medicines needed on a regular basis [7]. Currently, the FDA lists 206 discontinuations many of which can be easily substituted by the corresponding compounded medicines. Examples of these discontinuations include budesonide and ursodiol capsules [48]. Compounded formulas for the corresponding capsules and/or oral liquids are thoroughly referenced in the published literature (selected formulas are shown in Table 1).

#### 3.3.3. Economic Considerations

There are important economic considerations to take into account when preparing compounded medicines. The cost of these medicines is country specific as it depends on numerous factors, including the regulatory framework discussed in Section 2. Often, compounded medicines are more expensive than mass-produced proprietary medicines because pharmaceutical compounding is patient specific. However, there are situations where compounded medicines are significantly less expensive than the corresponding proprietary medicines. This is when ethical considerations ignite the much-debated topic of exorbitant pharmaceutical prices versus universal access to essential medications [49]. Compounded medicines should be a therapeutic option only when there are no equivalent commercial medicines available. However, exceptions should apply for the benefit of all patients.

## 4. The Impact of Compounding on Medication Adherence

When there is no therapeutic alternative provided by the pharmaceutical industry, fulfilling the unmet needs of patients is the primary aim of compounding. The promotion of adherence to medication represents a secondary outcome of pharmaceutical compounding in such a scenario. Another perspective is the impact of compounding on medication adherence in comparison with industrial medicines, particularly by comparing adherence rates between patients under treatment with compounding formulations and patients using proprietary medicines for the treatment of the same disease. Several assumptions can be proposed regarding the putative role of compounding in promoting medication adherence. The customization might represent a positive reinforcement of the initiation of the treatment, by offering patients the opportunity to be involved in making decisions about medicines and increasing their confidence in the efficacy of such tailored treatment. Patients involved in the selection of the treatment are usually more adherent [55]. Implementation might also be favored, since education about the posology and administration, and clarification of doubts can be easily achieved in a pharmacy setting. Regarding persistence, the usually short beyond-use dates of compounding formulations require regular refills and thus more visits to the pharmacy, paving the way for the role of pharmacists in educating the patient about the benefits of proper medication adherence. One constraint to the use of compound medications is the cost, which might represent a barrier to optimal adherence [56]. However, compounded medicines are at times cheaper than the corresponding commercial alternatives. When patients cannot afford the commercially available medicines, compounding may be the only alternative.

Besides these assumptions, studies addressing the influence of compounding in adherence promotion are lacking in the literature. Probably, the low extent of compounding medications gives rise to methodological issues and could have constrained the research on this topic. Compounded medications may also not be listed in e-prescribing software [57], compromising data collection and analysis. There are few reliable published data on the extent of compounding by community pharmacists and on the proportion of patients who use compounded medications. Most of the studies report that the prevalence of compounding practice is very low (less than 5%) [58]. The prevalence of compound users represented only 1.4% of eligible members (insured) in a study conducted in the USA in 2013 [56]. Several reasons might be appointed for the under exploration of the value of pharmaceutical compounding to public health, such as concerns about cost, potential risks, and compliance with good compounding practices. Compounding errors have been recently reviewed. Although compounding errors were identified in only 3% of the screened reports, most of them caused harm to patients [14]. A low prescription rate is also a major reason for the low prevalence of compounding. Prescriptions of compounding formulations represented 2.3% of all prescriptions dispensed by USA compounding pharmacies in 2006 [59]. This is more surprising when considering that the opportunity to benefit from this customized treatment seems to be available to most patients, although could be country specific. In a survey conducted in 2012, 85.5% of community pharmacies in the USA reported providing compounding services [60]. This scenario claims to the involvement of policymakers in providing a regulatory framework favorable to the compounding practice, beneficial both to pharmacies, patients, and public health.

**Figure 3:**
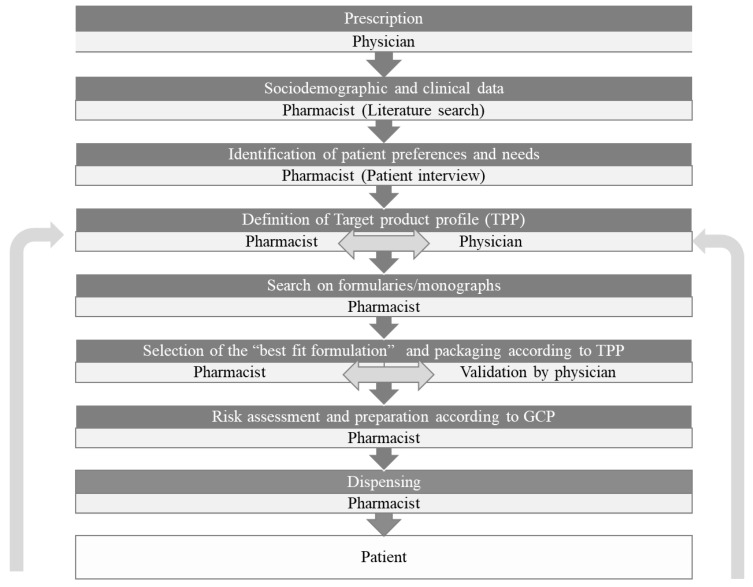
The process of patient-centric drug product design (PCDPD) applied to compounding formulations: Patient-centric compounding design (PCCD).

The process of patient-centric pharmaceutical drug product design (PCDPD) that identifies the comprehensive needs and preferences of individuals and uses this information to guide the design of pharmaceutical drug products can be highly valuable for the preparations prepared extemporaneously and tailored for a given patient. Whilst PCDPD is useful for pharmaceutical industries, especially when a significant body of data is available for a target population, it is streamlined when addressing individual patients. The contribution of PCDPD to the improvement of medication adherence has also been recently reviewed [6]. We propose a methodology of patient-centric compounding design (PCCD) to systematize the formulation of compounding medicines aiming to improve medication adherence (Figure 3). The data available for the population that is relevant according to the clinical condition and sociodemographic features of the patient starting a therapeutic regimen with a compounded formulation should be previously analyzed. Then, the patient should be interviewed to assess self-reported needs and preferences. Standard questionnaires would be very useful for harmonization. A target product profile (TPP) can then be defined taking into account the collected information by the pharmacist and the physician. The compounding pharmacist can select the “best fit formulation” and packaging by discussion with the prescribing physician after searching in official formularies and monographs. In every case, the quality and safety should be assured by performing risk assessments and using good compounding practices [16,18]. Insights provided by the patient after initiation can also be helpful in the optimization of TPP. This stepwise approach depicted in Figure 3 could represent a relevant pharmaceutical intervention to improve medication adherence.

## 5. Conclusions

Pharmaceutical compounding is still an important component of pharmacy practice even though several studies have underlined its low prevalence. Its value to patient care and public health could be increased in modern health care systems. We propose a methodology of patient-centric compounding design (PCCD) to systematize the formulation of compounding medicines aiming to improve medication adherence. Nevertheless, the putative contribution of compounding to the promotion of adherence to medication has not yet been explored. The results of such studies could support health policies including proper regulatory framework, pharmacist training, and information to health care practitioners.

## Figures and Tables

**Table 1:** Examples of compounded medicines and corresponding formulas.

Compounded Medicines	Formulas
Hydroxychloroquine sulfate 25 mg/mL oral suspension (adapted from [50])	Hydroxychloroquine sulfate.............................. 2.5 g Vehicle: Ora-Plus^®^ and Ora-Sweet^®^ SF (1:1) to 100 mL
Stanford modified oral rinse (adapted from [51])	Diphenhydramine hydrochloride........................ 0.0625 g Lidocaine hydrochloride monohydrate............ 0.533 g Nystatin (2,500,000 units)................................... 0.407 g Magnesium hydroxide..................................... 1 g Aluminum hydroxide gel .................................... 1 g Steviol glycosides 95%............................................. 0.1 g Acesulfame potassium FCC............................ 0.1 g Simethicone ............................................... 0.1 g Flavor, Crème DeMenthe ............................ 0.2 mL Purified water............................................ 15 mL Base, PCCA Mucolox™ .......................... to 100 mL
Transdermal pain medication (adapted from [36])	Ketamine hydrochloride.................................. 5 g Gabapentin.................................................. 10 g Clonidine hydrochloride............................... 0.2 g Baclofen....................................................... 2 g Ethoxy diglycol............................................. 10 g Base, PCCA Lipoderm^®^............................. to 100 g
Budesonide 1 mg/10 mL oral suspension (adapted from [52])	Budesonide................................................ 0.01 g Steviol glycosides 95%............................................. 0.2 g Parabens preserved water............................ 20 mL Base, PCCA Mucolox™ ............................ to 100 mL
Ursodiol 150 mg capsules (adapted from [53])	Ursodiol...................................................... 15 g Starch..........................................................to 100 capsules
Ursodiol 50 mg/mL oral suspension (adapted from [54])	Ursodiol....................................................... 25 g Acesulfame potassium .................................. 2.5 g Steviol glycosides 95%.............................................. 2.5 g Base, PCCA Suspendit^®^........................... to 500 mL
Anti-gag lollipops (adapted from [12])	Sodium chloride......................................... 46.56 g Potassium chloride......................................... 3 g Calcium lactate............................................. 6.12 g Magnesium citrate........................................ 2.04 g Sodium bicarbonate..................................... 22.44 g Sodium phosphate monobasic........................... 3.84 g Silica gel...................................................... 3.60 g Polyethylene glycol 1450.....................qs 36 lollipops

## Data Availability

Data sharing not applicable.
